# Carnitine Deficiency in Intensive Care Unit Patients Undergoing Continuous Renal Replacement Therapy: A Single-center Retrospective Study

**DOI:** 10.31662/jmaj.2023-0112

**Published:** 2023-11-16

**Authors:** Marina Oi, Takaaki Maruhashi, Yasushi Asari

**Affiliations:** 1 Department of Emergency and Critical Care Medicine, Kitasato University School of Medicine, Sagamihara, Japan

**Keywords:** Carnitine deficiency, Intensive care unit, High-volume hemofiltration, Continuous renal replacement therapy

## Abstract

**Introduction:**

Carnitine deficiency is common in patients undergoing intermittent hemodialysis and may also occur during continuous renal replacement therapy (CRRT). We evaluated intensive care unit (ICU) patients undergoing CRRT for carnitine deficiency and its associated risk factors.

**Methods:**

This was a single-center, retrospective, observational study performed between June 2019 and March 2020. The primary outcome was the incidence of carnitine deficiency in ICU patients undergoing CRRT.

**Results:**

Eighty-eight patients underwent 103 blood carnitine concentration measurements. The median age was 68 years (interquartile range: 55-80), Acute Physiology and Chronic Health Evaluation II score was 28 (24-33), Sequential Organ Failure score was 8.5 (5-11), Nutrition Risk in Critically Ill score was 6 (5-7), and blood carnitine concentration was 66.1 μmol/L (51.8-83.3). In total, 34 of 88 patients (38.6%) were found to have carnitine deficiency; however, there was no significant difference in the proportions of patients with carnitine deficiency characterized by disease. CRRT was performed in 44 (50%) patients, and the median blood total carnitine concentration measured after 24 h of CRRT without changing the settings was 65.5 μmol/L (48.6-83.3). The purification volume of CRRT and blood carnitine concentration were negatively correlated (R = −0.63; P = 0.02).

**Conclusions:**

Carnitine deficiency is seen in patients receiving CRRT and may increase in incidence as the purification volume increases, requiring regular monitoring.

## Introduction

Carnitine facilitates energy production through the transport of long-chain fatty acids into the mitochondria and the removal of waste metabolites ^(1)^. Approximately 98% of the total carnitine in the human body is stored in skeletal muscles and is transported by blood ^(2)^. Carnitine is mainly synthesized in the liver and kidneys from lysine and methionine. The amount of carnitine synthesized in the body is low, and the body maintains its concentration through reabsorption in the kidneys and storage in organs, such as the skeletal muscles, liver, kidneys, and heart ^(3)^. Exogenous carnitine supplementation is considered necessary when fatty acid demand is high, when the body’s stores are depleted ^(4)^, and in conditions such as severe trauma, sepsis, multiple organ failure, and malnutrition1. Approximately 95% of patients undergoing hemodialysis are carnitine-deficient, owing to extracorporeal loss of carnitine via dialysis and impaired renal carnitine synthesis ^(5)^. L-carnitine replacement therapy can reduce the risk of cardiac complications, anemia, muscle weakness, and other complications ^(6), (7), (8)^. However, reports on the comprehensive evaluation of carnitine concentrations in critically ill patients are scarce, and carnitine deficiency in intensive care settings has not been adequately assessed ^(9)^. Furthermore, reports of carnitine deficiency in adult patients undergoing continuous renal replacement therapy (CRRT), which is used in intensive care settings, are lacking. Symptoms of carnitine deficiency, such as anemia and muscle weakness, are important in intensive care settings and require treatment ^(10)^. Furthermore, although survival rates for sepsis have improved owing to advances in treatment ^(11)^, the subsequent motor dysfunction associated with muscle weakness (intensive care unit [ICU]-acquired weakness [ICU-AW]) is an ever-increasing clinical problem ^(12)^. Considering that sepsis is a common cause of admission to the ICU, it is of great clinical significance to understand the pathogenesis of carnitine deficiency in critically ill patients and evaluate its risk factors, prognosis, and association with CRRT, which have not been studied yet.

In this study, we aimed to examine carnitine deficiency in ICU patients undergoing CRRT and determine its associated risk factors, which were assumed to be the effects of the disease and CRRT.

## Materials and Methods

### Study design and patients

**Figure 1.**
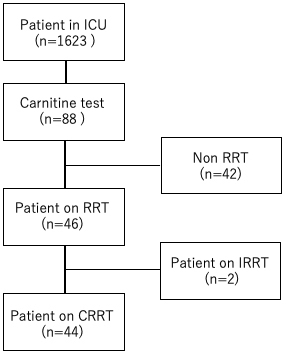
Patient flowchart During the study period, of the 1,623 patients admitted to the ICU, 44 underwent CRRT. Patients who did not receive RRT and those who received IRRT were excluded. RRT: renal replacement therapy, CRRT: continuous renal replacement therapy, IRRT: intermittent renal replacement therapy, ICU: intensive care unit.

This was a single-center, retrospective, observational study. Adult patients aged ≥20 years who were admitted to our center’s ICU between June 1, 2019, and March 31, 2020, were included (Figure 1). Carnitine measurements were performed in all patients who underwent CRRT in the ICU during the study period, as well as in patients who did not undergo CRRT but had concerns about carnitine deficiency in the context of low nutrition or severe sepsis. The decision to measure carnitine in patients who are not under-CRRT was left to the attending physician. Patients aged <20 years and those admitted to the ICU after a hospital emergency or scheduled surgery were excluded from the study. In ICU patients who underwent CRRT, blood carnitine concentrations were measured 24 h after commencing the therapy. There were no specific criteria for measuring blood carnitine concentrations, but it was at the discretion of the attending physicians on the basis of the patient’s admission to the ICU, nutritional needs, and other factors. The following data were also collected from the medical records: CRRT status, hemodialysis information (purification volume, types of dialysis membranes, and duration of dialysis), primary disease, duration of stay in the ICU (days), hematology data (arterial blood gas findings, complete blood counts, total bilirubin, creatinine, electrolyte, and C-reactive protein levels), Acute Physiology and Chronic Health Evaluation (APACHE) score, Sequential Organ Failure Assessment (SOFA) score, Nutrition Risk in Critically Ill (NUTRIC) score, body mass index (BMI), and outcomes.

### Definition of carnitine deficiency

In this study, carnitine deficiency was defined as either or both a free carnitine concentration below 36 μmol/L and an acylcarnitine to free carnitine ratio above 0.4, as published in the guidelines of the Japanese Pediatric Society ^(13)^.

### Carnitine concentration measurement

Blood carnitine concentrations were measured using an enzymatic cycling method by an independent laboratory outside of our institution (SRL Inc., Tokyo, Japan). Metabolically, L-carnitine is oxidized by carnitine dehydrogenase and thionicotinamide adenine dinucleotide (thio-NAD) to produce dehydrocarnitine and the reduced form, β-thionicotinamide adenine nucleotide (thio-NAD+). Subsequently, dehydrocarnitine generates L-carnitine and thio-NAD+ in the presence of carnitine dehydrogenase and reduced nicotinamide adenine dinucleotide (NADH). The concentration of free carnitine in a sample is proportional to the rate of formation of thio-NADH, which means that it can be measured by measuring the change in absorbance of thio-NADH.

### Laboratory measurements and survival

In patients undergoing CRRT, blood carnitine concentrations were measured 24 h after admission to the ICU, after the CRRT settings were maintained for at least 24 h. Carnitine concentrations were re-measured when CRRT settings were changed or when new disease-related complications occurred. BMI, SOFA, APACHE II, and NUTRIC scores were evaluated upon admission in the ICU. Survival was defined as the number of patients who were discharged from the hospital alive, and the survival rate was defined as the percentage of patients who were discharged alive.

### Outcomes

The primary outcome was the incidence of carnitine deficiency in patients undergoing CRRT. Secondary outcomes were the incidence of carnitine deficiency in each disease and the correlation between creatine kinase (CK) and carnitine fraction levels. Possible risk factors were age, sex, primary disease, dialysis status, and BMI, as well as SOFA, APACHE II, and NUTRIC scores.

### Statistical analysis

Multiple logistic regression analysis was performed to evaluate the possible risk factors. The correlation between CRRT blood purification volume and blood carnitine concentration was also evaluated. Numerical data are presented as n (%), and continuous data are presented as median (interquartile range [IQR]). SPSS version 21 (IBM Corp., Armonk, NY, USA) was used to analyze the data. P-values <0.05 were considered statistically significant.

### Ethics approval

The study protocol was approved by the Kitasato University Hospital Ethics Committee (Approval Number: B19-366). The need for informed consent was waived by the committee owing to the retrospective observational design of the study.

## Results

**Figure 2.**
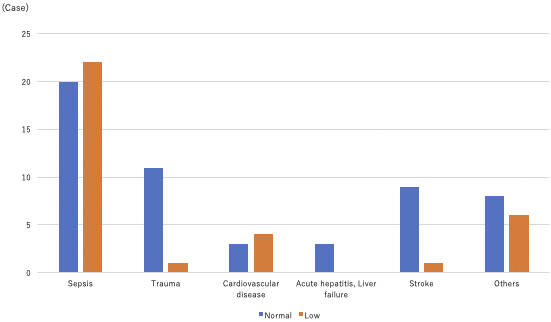
Blood carnitine concentration by disease The percentage of patients with carnitine deficiency for each disease is shown. Other diseases were anorexia nervosa and diabetic ketoacidosis, suggesting the possibility of background malnutrition.

A total of 103 blood carnitine concentration measurements were performed in 88 patients during the study period. The average follow-up period was 28 days. The patients’ median age was 68 (IQR: 55-80) years, with a male-to-female ratio of 1:1 (n = 88). The reasons for admission were sepsis, trauma, stroke, cardiac disease, acute hepatitis and/or liver failure, and other diseases (Table 1), with sepsis being the most common cause of carnitine deficiency. The median blood carnitine concentration was 66.1 (IQR: 51.8-83.3) μmol/L, and carnitine deficiency was observed in 34 patients (38.6%), with sepsis being the most common disease (22/42, 52.4%; Figure 2). No significant differences were observed in the percentage of patients with carnitine deficiency for each disease. The patients’ median (IQR) BMI is shown in Table 1.

**Table 1.** Characteristics of All ICU Patients with Carnitine Data and the Subset Who Underwent CRRT.

Characteristic	Median or n	IQR or %
**ICU patients with carnitine data**	88	
Age (years)	68	55-80
Sex (M:F)	44:44	
BMI	23.3	19.5-25.8
Diagnosis		
Sepsis	42	47.7
Trauma	12	13.6
Stroke	10	11.4
Cardiovascular disease	7	8
Acute hepatitis, liver failure	3	3.4
Others	14	15.9
APACHE Ⅱ score	28	24-33
SOFA score	8.5	5-11.1
NUTRIC score	6	5-7
ICU^*^ stay duration (days)	11	6-18.1
Survival to discharge	66	75%
**ICU patients with carnitine data who underwent CRRT**	44	
Reason for initiating CRRT		
AKI	32	72.7
CKD	3	6.8
Sepsis	4	9.1
Metabolic acidosis	2	4.5
Hyperkalemia	1	2.3
Congestive heart failure	2	4.5
Dialysis membrane		
AN69ST	35	79.6
PMMA	2	4.5
PS	7	15.9

IQR: interquartile range, M: male, F: female, BMI: body mass index, APACHE: Acute Physiology and Chronic Health Evaluation, SOFA: Sequential Organ Failure Assessment, NUTRIC: Nutrition Risk in Critically Ill, ICU: intensive care unit, CRRT: continuous renal replacement therapy, AKI: acute kidney injury, CKD: chronic kidney disease, AN69ST: acrylonitrile-co-methallyl sulfonate surface-treated, PMMA: polymethyl methacrylate, PS: polysulfone

The results of the multiple regression analysis of the possible risk factors showed no significant association between any of the risk factors and carnitine deficiency (data not shown). There was also no correlation between blood CK and blood total and free carnitine levels (R = 0.018 and R = 0.024, respectively), although CK was higher in patients who met the definition of carnitine deficiency (median 407 vs. 183.5: P < 0.05).

**Figure 3.**
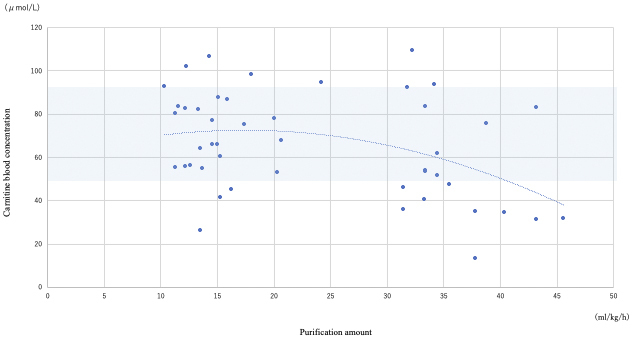
Relationship between blood purification volume and blood carnitine concentration in patients who underwent CRRT. The normal blood concentration for total carnitine is 45-91 μmol/L. As blood purification volume increased, blood carnitine concentration decreased. CRRT: continuous renal replacement therapy.

CRRT was performed in 44/88 (50%) patients; all 88 patients underwent blood carnitine concentration measurements. The median age of patients who underwent CRRT was 69 (IQR: 57-78) years, and the male-to-female ratio was 29:15. The primary diseases (i.e., the reasons for admission to the ICU) were sepsis, trauma, cardiac disease, acute hepatitis and/or liver failure, and other diseases in 28, 2, 5, 2, and 7 patients, respectively. The median APACHE II, SOFA, and NUTRIC scores were 31 (IQR: 27-35.3), 11 (IQR: 9-14), and 6 (5-7), respectively. Acute kidney injury was the most common indication for CRRT (n = 32; 72.7%). The median blood carnitine concentration was 65.5 (IQR: 48.6-83.4) μmol/L, and carnitine deficiency was present in 23/44 patients (52.3%). There was a negative correlation between CRRT blood purification volume and blood carnitine concentration (R = −0.63, P = 0.02; Figure 3).

## Discussion

In this study, carnitine deficiency was identified in 27% of the patients admitted to the ICU who had their blood carnitine concentrations measured; however, there was no significant difference in the proportions of patients with carnitine deficiency by disease. Although there was no statistically significant difference, carnitine deficiency was more commonly found in patients with sepsis. The link between carnitine deficiency and sepsis was first reported in 1989, and carnitine deficiency is believed to be caused by inhibition of the activity of enzymes involved in the l-carnitine-mediated transport of long-chain fatty acids from the cytosol to the mitochondria during the β-oxidation process of fatty acids ^(14)^. Clinical trials have since reported positive results for l-carnitine supplementation in patients with sepsis ^(15)^. Meanwhile, the participants in these studies were not found to be carnitine-deficient in blood tests, and it is debatable whether carnitine is relatively or absolutely deficient in sepsis. Although relative carnitine deficiency has been noted in sepsis ^(16)^, the present study also found absolute carnitine deficiency in patients with sepsis, suggesting that this may be of additional importance with regard to replacement therapy.

However, additional data and analyses are needed to determine the effect of each disease on carnitine deficiency. Notably, an association was found between blood carnitine levels and CRRT, suggesting a trend toward an increased risk of carnitine deficiency as the amount of CRRT purification volume increases.

Carnitine is a small molecule with a molecular weight of 161 Da, and its blood concentration decreases by 60%-70% after each hemodialysis session ^(17)^. Carnitine deficiency has been confirmed in patients undergoing maintenance hemodialysis ^(15)^. The basic principle of hemodialysis is similar to that of CRRT; however, the blood purification volume is smaller with CRRT than with maintenance dialysis because CRRT does not use a dialysis shunt with an anastomotic arteriovenous connection. Furthermore, CRRT is not used long term as with maintenance dialysis since most patients requiring CRRT have acute conditions, such as acute kidney injury ^(18)^. Considering these characteristics, the incidence of carnitine deficiency in patients undergoing CRRT is expected to be lower than that in patients undergoing maintenance dialysis; however, this has not yet been studied. Notably, there are cases in which CRRT is performed at high-volume hemofiltration rates in the ICU owing to critical illness, and there are also cases in which CRRT must be performed long term owing to an unstable disease.

It is important to understand the incidence of carnitine deficiency in patients undergoing CRRT, as previous studies in pediatric patients undergoing CRRT reported that 100% of the patients undergoing CRRT for more than 3 weeks developed carnitine deficiency ^(17)^. Carnitine deficiency results in the inability of the body to oxidize fatty acids, resulting in various symptoms such as muscle weakness, rhabdomyolysis, cardiomyopathy, and arrhythmias ^(4)^. In our study, carnitine deficiency was observed in 52.3% of the patients who underwent CRRT and had carnitine measurements at the time of inclusion in the study, with a trend toward increasing carnitine deficiency as the dialysis flow rate increased. Regular follow-up of blood carnitine levels was considered necessary in patients who undergo continuous high-flow dialysis.

ICU-AW, which is a motor dysfunction associated with muscle weakness in ICU patients, is an important disease that determines patient prognosis, and there is no established effective treatment. Sepsis, which is considered a risk factor for ICU-AW, is one of the primary diseases for which CRRT is commenced in the ICU ^(19)^; it accounted for half of the eligible ICU cases in this study. Furthermore, recent reports have found that muscle weakness in ICU patients is due to mitochondrial dysfunction ^(9), (20)^. Impaired β-oxidation of fatty acids in the mitochondria may induce the accumulation of acyl-CoA and contribute to mitochondrial dysfunction ^(9), (21)^. Carnitine supplementation, which is involved in acyl-CoA metabolism, may be a treatment for ICU-AW ^(9), (21)^. In addition, animal studies have reported that muscle weakness after sepsis is exacerbated by mitochondrial dysfunction ^(22)^, and carnitine deficiency is a risk factor for ICU-AW4. Although the Medical Research Council Score (the diagnostic criterion for ICU-AW) was not measured in all patients in this study, approximately 40% of the patients who underwent CRRT had muscle weakness to the extent that they had difficulty standing when leaving the ICU. Further investigation of the efficacy of carnitine replacement therapy as a method of prevention and treatment of ICU-AW is warranted.

In terms of limitations, this was a single-center, retrospective, observational, pilot study with a limited number of eligible patients. This may have resulted in a bias in the diseases and severity of illnesses that resulted in hospitalization. Additionally, the evaluation of ICU-AW was limited because it was performed only during hospitalization, and we did not follow-up the patients in the long term. The direct relationship between carnitine concentration and the outcomes is also unclear and needs to be validated in a prospective study involving patients who are identified as carnitine-deficient undergoing replacement therapy. In addition, since the carnitine fraction was not measured prior to the start of CRRT, no assessment could be made regarding a potential carnitine deficiency.

Although studies have evaluated the development of carnitine deficiency in patients undergoing hemodialysis and the effect of replacement therapy in these patients, these concerns have not been studied in patients undergoing CRRT. Our results showed that carnitine deficiency may occur in patients undergoing CRRT, and the risk increases as the flow rates increase. The efficacy of replacement therapy for carnitine deficiency in patients undergoing CRRT should be investigated in future studies.

## Article Information

### Conflicts of Interest

None

### Acknowledgement

The authors would like to thank Editage (https://www.editage.jp) for the English language review. We thank Jane Charbonneau, DVM, from Edanz (https://jp.edanz.com/ac) for editing the draft of this manuscript.

### Author Contributions

MO designed the study and drafted the manuscript. TM conducted statistical analysis. YA critically revised the manuscript for important intellectual content. All authors read and approved the final manuscript. All authors agree to be accountable for all aspects of the work and for ensuring that questions related to the accuracy or integrity of any part of the work are appropriately investigated and resolved.

### Approval by Institutional Review Board (IRB)

The study protocol was approved by the Kitasato University Hospital Ethics Committee (Approval Code: B19-366).

### Data Availability

The datasets used or analyzed during the current study are available from the corresponding author on reasonable request.
